# Epoetin beta pegol for treatment of anemia ameliorates deterioration of erythrocyte quality associated with chronic kidney disease

**DOI:** 10.1186/s12882-018-0818-4

**Published:** 2018-01-27

**Authors:** Ken Aizawa, Ryohei Kawasaki, Yoshihito Tashiro, Yasushi Shimonaka, Michinori Hirata

**Affiliations:** Product Research Department, Chugai Pharmaceutical Co., Ltd, 200 Kajiwara, Kamakura, 247-8530 Japan

**Keywords:** Epoetin beta pegol (C.E.R.A.), Chronic kidney disease (CKD), Anemia, Hematopoiesis, Erythrocyte deformability, Erythrocyte turnover, Intracellular calcium

## Abstract

**Background:**

Epoetin beta pegol (continuous erythropoietin receptor activator; C.E.R.A.) is currently widely used for the treatment of anemia associated with chronic kidney disease (CKD). Therapeutic control of anemia is assessed by monitoring haemoglobin (Hb) levels. However, certain qualitative aspects of erythrocytes are also impaired in CKD, including loss of deformability and shortened life-span. Therefore, monitoring Hb alone could potentially fail to reveal pathological changes in erythrocytes. Focusing on erythrocyte quality in CKD may lead to more effective anemia therapy with C.E.R.A.

**Methods:**

A CKD rat model was induced by uninephrectomy followed by anti-Thy1.1 antibody injection. From 5 weeks after the operation, C.E.R.A. (0.6 μg/kg) or vehicle was administered every 2 weeks. Erythrocyte deformability was quantified with ektacytometry and erythrocyte turnover was estimated by biotin labeling. Intracellular calcium level was assessed by Fluo-3/AM.

**Results:**

Erythrocyte deformability progressively declined in CKD rats. Furthermore, erythrocyte turnover in the circulation drastically accelerated in CKD rats. With administration of C.E.R.A. at a dose sufficient to adequately control Hb, deterioration of erythrocyte deformability and turnover in CKD rats were significantly improved. Intracellular calcium, which plays a pivotal role in the mediation of erythrocyte quality, was significantly increased in CKD and was normalized by C.E.R.A. treatment.

**Conclusion:**

C.E.R.A. treatment exerted a favorable effect not only on anemia but also on the improvement of erythrocyte quality. C.E.R.A. administered for the treatment of CKD-associated anemia may confer therapeutic benefits on erythrocytes.

## Background

Anemia is a complication of chronic kidney disease (CKD) that arises due to the malfunction of erythropoietin (EPO) production as kidney function declines. Therefore, supplementation with EPO can be a highly effective form of therapy. Several erythropoiesis-stimulating agents (ESAs) play an indispensable role in clinical practice for the treatment of renal anemia. Among these is epoetin beta pegol (continuous erythropoietin receptor activator; C.E.R.A.), a modified EPO which has the longest half-life of all ESAs in the circulation of CKD patients [1].

However, it has been suggested that in CKD patients not only does the number of erythrocytes decrease (anemia) but also that there is some deterioration in erythrocyte quality. For example, a loss of deformability [2, 3] and a shortened life-span [4–6] can be observed in erythrocytes from CKD patients. The diameter of the peripheral arteries is 5 to 10 μm, whereas the diameter of a normal erythrocyte is approximately 8 μm [7]. Therefore, erythrocyte deformability is a necessity to allow the normal passage of erythrocytes through the microcirculation capillaries so that they can play their vital roles such as oxygen delivery. In addition, low erythrocyte deformability itself can reduce the degree of oxygen uptake and donation [8]. It has also been suggested that deterioration of erythrocyte quality is associated with poor prognosis in CKD [9] and with severity of hypertension [10]. Therefore, monitoring the qualitative aspects of erythrocytes in CKD patients is clinically important, and will contribute to providing advantageous therapeutic benefits to overcome anemia. However, little attention has been paid to erythrocyte quality in the clinical setting, possibly because treatment of anemia with ESAs easily enables control of haemoglobin (Hb) levels. Nevertheless, merely ensuring appropriate Hb levels may not be sufficient to provide the most effective treatment of CKD-associated anemia.

Several studies have suggested that ESAs have a beneficial effect on erythrocyte quality in CKD [11, 12]. However, the mechanisms underlying deterioration of erythrocyte deformability and life-span have not yet been fully elucidated. In particular, it is not yet clear whether treatment of anemia with C.E.R.A. affects erythrocyte quality in CKD. In this study, we explored the favorable effects of C.E.R.A. on erythrocyte quality, including deformability and life-span, using uninephrectomized anti-Thy1.1 antibody-induced progressive glomerulosclerosis (UNX-Thy1) rats as a CKD model.

## Methods

### Experimental animals used for the CKD model

Male Fisher 344 rats (6 weeks old) were purchased from Charles River Japan (Yokohama, Japan) and allowed to acclimatize for 1 week. All rats were allowed free access to food and water. The CKD model was produced following the method reported previously [13]. Briefly, after left kidney nephrectomy, an anti-rat CD90 (Thy1.1) monoclonal antibody (Cedarlane Labs, Burlington, Canada) at a single dose of 0.6 mg/kg body weight was intravenously injected into rats (UNX-Thy1 group, *n* = 12). Sham-operated rats (Sham group, *n* = 5) underwent surgical manipulation without any removal of the kidney. Sham rats were injected with phosphate buffered saline (PBS) instead of the antibody. All animal procedures and experimental protocols were approved by the Institutional Animal Care and Use Committee at Chugai Pharmaceutical Co., Ltd., and conformed to the *Guide for the Care and Use of Laboratory Animals* published by Institution of Laboratory Animal Resources (ILAR).

### Treatments

After the UNX-Thy1 and sham operations, UNX-Thy1 rats were randomly assigned to two groups: a vehicle-treated group (UNX-Thy1 + Vehicle, *n* = 6) and a C.E.R.A.-treated group (UNX-Thy1 + C.E.R.A., *n* = 6). C.E.R.A. (obtained from Chugai Pharmaceutical Co., Ltd., Tokyo, Japan) was administered intravenously at 0.6 μg/kg every 2 weeks from 5 weeks after the UNX-Thy1 operation. The UNX-Thy1 + Vehicle group was administered PBS-Tween 80 (0.02%, *v*/v). For the analysis of Hb, mean corpuscular volume (MCV), red blood cell (RBC) count, and erythrocyte deformability, heparinized blood was drawn via the jugular vein of anesthetized rats every week except for Week 8. At the end of the experiment (Week 18), after the collection of 24-h urinary samples for the analysis of urinary total protein, the animals were anesthetized under isoflurane and euthanized by exsanguination from the abdominal aorta and the blood was collected for the analysis of plasma creatinine and plasma calcium. Body weight (BW), spleen weight/body weight ratio (SW/BW), and heart weight/body weight ratio (HW/BW) were also measured at the end of the experiment.

### Measurement of biological parameters

Hb, MCV, and RBC count were measured with an automated hematology analyzer (XT-2000iV; Sysmex, Hyogo, Japan). Plasma creatinine and urinary total protein were measured with an automatic analyzer (TBA-2000FR; Toshiba, Tochigi, Japan). Plasma calcium was measured with a Metallo Assay LS trace metal assay kit for calcium (CPZ III; MG Metallogenics, Chiba, Japan) according to the manufacturer’s protocol. To assess oxidative stress, the value of d-ROMs (derivatives of reactive oxygen metabolites) in the plasma was determined by FREE Carpe Diem (Diacron International, Grosseto, Italy), as described in a previous report [14].

### Erythrocyte deformability assay

Erythrocyte deformability was measured with an ektacytometer (LoRRca MaxSis; RR Mechatronics, Hoorn, The Netherlands). Briefly, whole blood was suspended in a dedicated isotonic viscous solution. A suspension of erythrocytes was introduced into a narrow space between two concentric glass cylinders and subjected to shear stress by centrifugal force created by rotation. Elongated erythrocytes produce a distinctive pattern of diffracted light. This diffraction pattern at 37 °C and at a fluid shear stress of 30 Pa was analysed and expressed as the elongation index (El), an index of erythrocyte deformability; EI = (*a* − *b*)/(*a* + *b*), where *a* and *b* are the length and width of the diffraction pattern, respectively. A higher EI indicates greater cell deformability.

### Assessment of erythrocyte turnover

At 12 weeks after the UNX-Thy1 operation, erythrocytes in circulation were biotinylated by tail vein injection of 3 mg EZ-Link Sulfo-NHS-Biotin (Thermo Fisher Scientific, Hanover Park, IL, USA) dissolved in 0.2 mL PBS [15]. At 1 to 6 weeks after the biotinylation (13 to 18 weeks after the UNX-Thy1 operation), blood collected from the jugular vein was washed in PBS supplemented with 2% heat-inactivated fetal bovine serum (FBS) and labeled with Streptavidin PE (1:200) (eBioscience, San Diego, CA, USA) for 30 min under protection from light, and then analysed by flow cytometry (LSR Fortessa X-20; BD BioSciences, San Jose, CA, USA). The ratio of biotinylated erythrocytes per net amount of analysed erythrocytes (100,000 cells) was quantified. The total number of biotinylated erythrocytes in the rats was estimated from RBC count and the ratio of biotinylated erythrocytes. Total bilirubin in plasma at Week 18 was assessed with an ELISA kit (MyBioSource, San Diego, CA, USA) according to the manufacturer’s protocol.

### Assessment of erythrocyte intracellular calcium

Intracellular calcium level of erythrocytes was assessed with Fluo-3/AM, a membrane-permeable calcium ion indicator (Calbiochem, San Diego, CA, USA). Blood collected from the jugular vein at Week 18 was washed in PBS/2% FBS, incubated with Fluo-3/AM (8 μM in PBS supplemented with 10 mM D-glucose) for 30 min at 37 °C under protection from light, and then analysed by flow cytometry [16].

### Statistical analysis

All values are shown as mean ± SEM. Statistical analysis was performed using JMP software (SAS Institute, Cary, NC, USA). Intergroup comparisons were assessed by two-way analysis of variance (ANOVA) followed by Tukey’s test for time-course data (Week 5 to Week 18) or Turkey’s test to compare three groups. Statistical analysis for correlation was assessed by Pearson’s correlation test. A value of *P* < 0.05 was considered statistically significant.

## Results

### Therapeutic effect of C.E.R.A. on anemia in CKD model rats

**Fig. 1:**
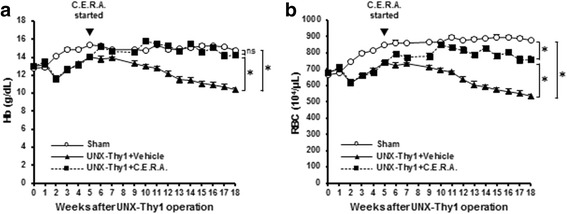
Time course of Hb and RBC count. Hb levels (**a**) and RBC numbers (**b**) progressively and significantly decreased in the UNX-Thy1 + Vehicle group compared with levels in the Sham group. **a** C.E.R.A. treatment in the UNX-Thy1 group significantly increased Hb to levels nearly the same as those in the Sham group. **b** RBC number was also significantly increased by C.E.R.A. treatment in the UNX-Thy1 group. Values are shown as mean ± SEM. *, *P* < 0.05 (**a**, **b**, two-way ANOVA followed by Tukey’s test). Sham, *n* = 5; UNX-Thy1 + Vehicle, *n* = 6; UNX-Thy1 + C.E.R.A., *n* = 6

Compared with Hb levels and RBC numbers in the Sham group, Hb levels and RBC numbers decreased at 2 weeks after the UNX-Thy1 operation, recovered slightly until Week 5, and then progressively decreased until the end of the experimental period (Hb, Fig. 1a; RBC, Fig. 1b). C.E.R.A. treatment from Week 5 significantly improved the anemic state of UNX-Thy1 rats. Hb levels were well controlled by C.E.R.A. treatment, and were at the nearly same level as in the Sham group by the end of the experiment (Fig. 1a). The time-course of RBC numbers was similar to that of Hb, although the RBC numbers in the UNX-Thy1 + C.E.R.A. group did not overlap those in the Sham group (Fig. 1b). At Week 18, MCV was significantly increased in the UNX-Thy1 + Vehicle group compared with MCV in the Sham group (Table 1). The significant increase in MCV was significantly decreased by C.E.R.A. treatment (Table 1). BW in the UNX-Thy1 + Vehicle group was significantly decreased compared to BW in the Sham group, and SW/BW and HW/BW in the UNX-Thy1 + Vehicle group were significantly increased compared with those in the Sham group (Table 2). C.E.R.A. treatment did not significantly affect these changes in BW, SW/BW, or HW/BW (Table 2).

**Table 1:** Biological parameters at Week 18

	Sham	UNX-Thy1 + Vehicle	UNX-Thy1 + C.E.R.A.
MCV (fL)	48.6 ± 0.1	53.8 ± 0.4*	52.1 ± 0.5*^,†^
Urinary total protein (mg/day)	4.4 ± 0.5	124.5 ± 16.3*	156.9 ± 15.0*
Plasma creatinine (mg/dL)	0.33 ± 0.01	3.04 ± 0.23*	2.62 ± 0.34*
Plasma calcium (mg/dL)	9.7 ± 0.2	9.6 ± 0.4	9.2 ± 0.2

Values are shown as mean ± SEM

*P < 0.05 vs. Sham; ^†^*P* < 0.05 vs. UNX-Thy1 + Vehicle (Tukey’s test)

Sham, *n* = 5; UNX-Thy1 + Vehicle, *n* = 6; UNX-Thy1 + C.E.R.A., *n* = 6

**Table 2:** Body compositions at Week 18

	Sham	UNX-Thy1 + Vehicle	UNX-Thy1 + C.E.R.A.
BW (g)	342.1 ± 4.0	236.6 ± 10.6*	246.2 ± 19.4*
SW/BW (mg/g)	1.93 ± 0.04	2.75 ± 0.04*	2.86 ± 0.06*
HW/BW (mg/g)	1.84 ± 0.03	2.35 ± 0.06*	2.28 ± 0.12*

*BW*, body weight, *SW/BW* spleen weight/body weight ratio, *HW/BW* heart weight/body weight ratio

Values are shown as mean ± SEM

*P < 0.05 vs. Sham (Tukey’s test)

Sham, *n* = 5; UNX-Thy1 + Vehicle, *n* = 6; UNX-Thy1 + C.E.R.A., *n* = 6

### Effect of C.E.R.A. on kidney protection in CKD model rats

To explore the effect of kidney protection by C.E.R.A. [13], we assessed several biological parameters. The levels of urinary total protein and plasma creatinine as parameters of kidney dysfunction were significantly increased in UNX-Thy1 + Vehicle rats compared with those in Sham rats (Table 1). In this experiment, C.E.R.A. treatment did not significantly change these indicators of kidney deterioration in UNX-Thy1 model rats (Table 1).

### Effect of C.E.R.A. on oxidative stress in CKD model rats

**Fig. 2:**
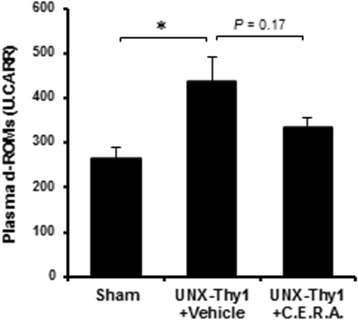
Plasma oxidative stress. The level of d-ROMs was significantly increased in the plasma of the UNX-Thy1 + Vehicle group compared with the Sham group. C.E.R.A. treatment in the UNX-Thy1 group tended to decrease the levels of plasma d-ROMs. Values are shown as mean ± SEM. *, *P* < 0.05 (Tukey’s test). Sham, *n* = 5; UNX-Thy1 + Vehicle, *n* = 6; UNX-Thy1 + C.E.R.A., *n* = 6

To explore the effect of C.E.R.A. on oxidative stress, we measured d-ROMs (an oxidative stress marker) in the plasma of the rats. A significant increase in d-ROMs was observed in the plasma of the UNX-Thy1 + Vehicle rats compared with that in the Sham rats. C.E.R.A. treatment tended to decrease the levels of d-ROMs in UNX-Thy1 model rats (Fig. 2).

### Effect of C.E.R.A. on erythrocyte deformability in CKD model rats

**Fig. 3:**
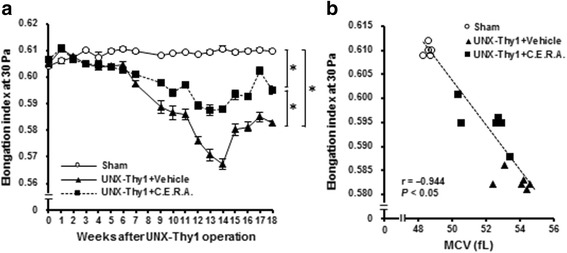
Erythrocyte deformability. Erythrocyte deformability is shown as the value of the elongation index. **a** Compared with the stable erythrocyte deformability status in the Sham group, erythrocytes in the UNX-Thy1 + Vehicle group showed a significant deterioration of deformability. C.E.R.A. treatment in UNX-Thy1 group significantly improved erythrocyte deformability. **b** A significant negative correlation was observed between MCV and erythrocyte deformability. Values are shown as mean ± SEM. *, *P* < 0.05 (**a**, two-way ANOVA followed by Tukey’s test; **b**, Pearson’s correlation test). Sham, *n* = 5; UNX-Thy1 + Vehicle, *n* = 6; UNX-Thy1 + C.E.R.A., *n* = 6

Erythrocyte deformability gradually declined after the UNX-Thy1 operation; the erythrocyte deformability (elongation index) was significantly lower in the UNX-Thy1 + Vehicle group than in the Sham group (Fig. 3a). On the other hand, C.E.R.A. treatment significantly improved the erythrocyte deformability in the CKD model rats (Fig. 3a). MCV showed a strong negative correlation with the elongation index at Week 18 (Fig. 3b).

### Effect of C.E.R.A. on erythrocyte turnover in CKD model rats

**Fig. 4:**
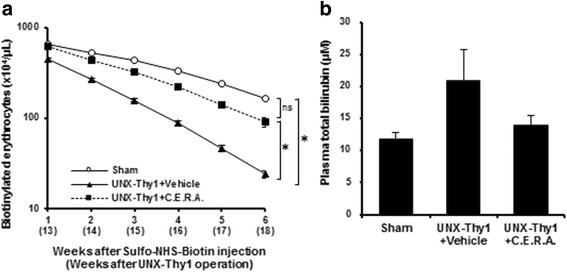
Erythrocyte turnover. **a** Biotinylated erythrocytes significantly decreased in the UNX-Thy1 + Vehicle group compared with in the Sham group. C.E.R.A. treatment in the UNX-Thy1 group significantly suppressed the decrease in biotinylated erythrocytes in circulation. **b** Although there was no statistical significance, total bilirubin in the plasma of the UNX-Thy1 + Vehicle group tended to increase compared with that in the Sham group. C.E.R.A. treatment in the UNX-Thy1 group tended to decrease plasma total bilirubin. Values are shown as mean ± SEM. *, *P* < 0.05 (**a**, two-way ANOVA followed by Tukey’s test; **b**, Tukey’s test). Sham, *n* = 5; UNX-Thy1 + Vehicle, *n* = 6; UNX-Thy1 + C.E.R.A., *n* = 6

To investigate whether therapeutic treatment of anemia with C.E.R.A. affected the life-spans of erythrocytes, the turnover of erythrocytes in the circulation of rats was estimated by the biotinylation method. At Week 12 after the UNX-Thy1 operation, erythrocytes in circulation at that time were labelled with Sulfo-NHS-Biotin [15]. Because of the increase in number of newly developed erythrocytes through hematopoiesis and because of the breakdown of old erythrocytes, the number of biotin-labeled erythrocytes time-dependently decreased in rats in the Sham group (Fig. 4a). Compared with the Sham group, the number of biotinylated erythrocytes markedly and quickly decreased in UNX-Thy1 + Vehicle rats (Fig. 4a). On the other hand, in CKD model rats treated with C.E.R.A., the decrease in number of biotinylated erythrocytes was significantly suppressed (Fig. 4a). To investigate the possibility of haemolysis, we assessed the level of total bilirubin in the plasma of rats. The plasma total bilirubin in UNX-Thy1 + Vehicle rats tended to increase but did not show any statistically significant difference compared with that in the Sham group (Fig. 4b). C.E.R.A. treatment tended to decrease the plasma total bilirubin although this was also not statistically significant (Fig. 4b).

### Effect of C.E.R.A. on erythrocyte intracellular calcium in CKD model rats

**Fig. 5:**
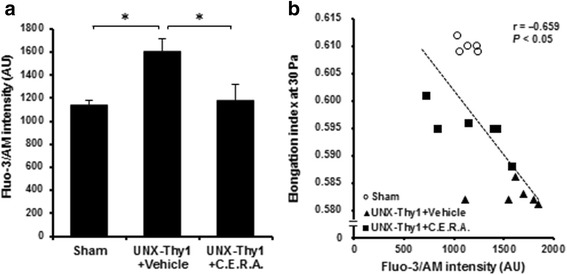
Intracellular calcium level in erythrocytes. **a** Intracellular calcium level as assessed by Fluo-3/AM intensity was significantly increased in erythrocytes in the UNX-Thy1 + Vehicle group compared with those in the Sham group. C.E.R.A. treatment in the UNX-Thy1 group significantly decreased intracellular calcium level to nearly the same level as in the Sham group. **b** A significant negative correlation was observed between intracellular calcium level in erythrocytes and erythrocyte deformability. Values are shown as mean ± SEM. *, *P* < 0.05 (**a**, Tukey’s test; **b**, Pearson’s correlation test). Sham, *n* = 5; UNX-Thy1 + Vehicle, *n* = 6; UNX-Thy1 + C.E.R.A., *n* = 6

To investigate the mechanisms underlying the changes in erythrocyte quality, we focused on intracellular calcium in erythrocytes. The level of intracellular calcium in erythrocytes in the UNX-Thy1 + Vehicle group was significantly increased compared with that in the Sham group (Fig. 5a). C.E.R.A. treatment significantly suppressed the level of intracellular calcium compared with that in the UNX-Thy1 + Vehicle group (Fig. 5a). There was no significant difference in the level of plasma calcium between any of the three experimental groups (Table 1). The level of intracellular calcium in erythrocytes showed a significant negative correlation with the elongation index at Week 18 (Fig. 5b).

## Discussion

This study provides the first evidence that adequate therapy with C.E.R.A. contributes not only to the treatment of renal anemia but also to the improvement of erythrocyte quality, including erythrocyte deformability and life-span. Although relevant reports are few, some previous studies have suggested that ESAs have beneficial effects on erythrocyte quality [11, 12], and moreover, that they are beneficial in decreasing left ventricular hypertrophy accompanying CKD [17]. However, it has not yet been clarified whether therapeutic treatment of anemia with C.E.R.A. affects erythrocyte quality in CKD. In our study, the deteriorations in erythrocyte deformability and life-span in CKD model rats were significantly ameliorated under conditions where Hb was controlled by treatment with C.E.R.A.

The biconcave discoid shape of erythrocytes allows the surface area to far exceed that of a sphere of the same volume. This enables normal erythrocytes to deform uniaxially while retaining a constant surface area as they pass through narrow capillaries. Disruption of the erythrocyte membrane structure induces membrane instability, resulting in morphological impairment. Morphological alteration is partly reflected by changes in MCV. Therefore, our present data regarding MCV may imply that morphological changes have occurred in erythrocytes in the CKD model rats. It has been suggested that erythrocytes in CKD patients tend to transform into a spherical shape via calcium signaling [18]. An increase in MCV may reflect the alteration of erythrocytes to a spherical shape, resulting in a loss of deformability [19, 20]. Indeed, in our experiment, the correlative analysis showed a strong negative relationship between MCV and erythrocyte deformability.

The membrane of erythrocytes contains a lipid bilayer which is similar to a soap bubble that cannot stretch. Therefore, to stabilize the membrane and provide an elastic biconcave shape that is resistant to deformation, the membrane is anchored by a filamentous network of proteins [21, 22]. The properties of membrane deformability and stability are principally regulated by membrane protein interactions [23]. Crosslinking between several membrane proteins, such as spectrin, ankyrin, and actin, establishes the skeletal dynamics which determines erythrocyte shape and deformability [21]. It is natural that severe deterioration of deformability through disruption of the erythrocyte membranous structure would lead to the shortening of erythrocyte life-span. Intriguingly, we could not detect any significant difference in the expression levels of typical membrane proteins, such as ankyrin, following UNX-Thy1 operation or C.E.R.A. treatment (data not shown, in-house data). It may be that erythrocyte deformability can be attributed to membrane structure rather than to the amounts of membrane proteins. Further research focusing on membrane structure will likely provide new insights.

It has been suggested that the structure and conformation of the erythrocyte membrane are dependent on the intracellular calcium ion concentration [24, 25]. Calcium signaling regulates membrane stability through modulation of skeletal protein interactions [24]. Previous studies have suggested that CKD is associated with unregulated intracellular calcium accumulation [18, 26, 27]. Increased levels of cytosolic calcium ions leads to stimulation of cell membrane scrambling with breakdown of phospholipid asymmetry of the cell membrane and phosphatidylserine exposure at the cell surface [28, 29]. An increase in MCV [25] and a decrease in erythrocyte deformability [24] can be also induced by calcium loading in erythrocytes. Therefore, in this study, we focused on and investigated whether the level of intracellular calcium in erythrocytes would be changed by CKD and C.E.R.A. treatment. Our present data clearly indicated that the level of intracellular calcium was increased in erythrocytes of CKD model rats and was suppressed by the therapeutic administration of C.E.R.A. In addition, there was a strong negative correlation between the level of intracellular calcium and erythrocyte deformability. These data suggested that intracellular calcium level played a crucial role in maintaining erythrocyte deformability. Although the upstream mechanisms mediating calcium signaling in CKD or following treatment with ESAs have not been elucidated, it is noteworthy that unselective calcium ion–permeable cation channels are inhibited by EPO which may thus counteract calcium entry [28]. Furthermore, the suppressive effect of EPO on erythrocyte calcium uptake has also been observed in CKD patients [27, 30]. Further research is still needed to explore the details of the underlying mechanism.

In this study, we estimated the turnover of erythrocytes by the biotinylation method. The time-course of the RBC numbers was similar to that of Hb, although the RBC numbers in the UNX-Thy1 + C.E.R.A. group did not overlap those in the Sham group, whereas the Hb curves in the Sham and UNX-Thy1 + C.E.R.A. groups did overlap. This difference may reflect the expansion of the extracellular volume in the UNX-Thy1 rat model. Actually, the MCV in the UNX-Thy1 + C.E.R.A. group was relatively higher than that in the Sham group even at the end point of the experiment, despite the significant decrease in MCV compared to that in the UNX-Thy1 + Vehicle group. Because the absolute number of RBCs was markedly increased by C.E.R.A. treatment, it seems to be natural that the slope of biotinylated RBC numbers observed in the UNX-Thy1 + C.E.R.A. group would be gentler than that in the UNX-Thy1 + Vehicle group. However, the decrease in the number of biotinylated RBCs was extremely rapid even after taking into consideration the change in total RBC number; whereas the difference in RBC numbers between the UNX-Thy1 + Vehicle group and the UNX-Thy1 + C.E.R.A. group was 1.4-fold at the point of the largest difference, the difference in biotinylated RBC numbers was nearly 3.8-fold. In addition, in the UNX-Thy1 + C.E.R.A. group, because the unlabeled RBCs were newly generated and were added into the already existing RBC population, the ratio of biotinylated RBCs was expected to be relatively decreased. In our assessment, we counted the number of biotinylated RBCs using the same number of RBCs and calculated the ratio of biotinylated RBCs. Nevertheless, the number of biotinylated RBCs was more rapidly decreased in the UNX-Thy1 + Vehicle group compared to that in the UNX-Thy1 + C.E.R.A. group. This was why we assumed that the elimination of erythrocytes that was accelerated in CKD rats could be prevented by the treatment of C.E.R.A.

The decrease in number of biotinylated erythrocytes reflects (1) the relative decrease due to the increase in non-labeled erythrocytes through hematopoiesis and (2) the elimination of erythrocytes by metabolism or destruction. Because hematopoiesis was attenuated in UNX-Thy1 + Vehicle rats, the increase of newly born erythrocytes would be lower than that in Sham rats; nevertheless, a quicker decrease in the number of biotinylated erythrocytes was observed in UNX-Thy1 + Vehicle rats. Consistent with the results of a clinical study [4], this suggested that elimination of erythrocytes from circulation (such as by haemolysis) was accelerated in the CKD model. This possibility is also supported by the tendency of plasma total bilirubin to increase. Despite there being no statistical difference, a slight decrease in biotinylated erythrocytes was seen in UNX-Thy1 + C.E.R.A. rats compared with Sham rats. It is plausible that the relative increase of newborn erythrocytes by C.E.R.A.-stimulated hematopoiesis may account for this slight decrease.

The accumulation of uremic toxins in circulation may also have an influence on erythrocyte quality [9, 31]. However, under our experimental conditions, the deterioration of erythrocyte quality appears to be caused by intrinsic factors rather than extracellular factors, because no significant effect on kidney protection was detected. Regarding this, we found no significant changes between HW/BW in the UNX-Thy1 + C.E.R.A. group and that in the UNX-Thy1 + Vehicle group, indicating that no tissue protective effect resulted from C.E.R.A. treatment at the dosage of C.E.R.A. used in this study. In addition, SW/BW was not significantly changed by C.E.R.A. treatment, which suggests that no drastic changes in extramedullary hematopoiesis or erythrocyte destruction in the spleen occurred under C.E.R.A. treatment. Compared with aged erythrocytes, young erythrocytes may be more stable and resistant to a cytotoxic environment [32]. Simply boosting hematopoiesis and increasing the number of young erythrocytes by ESA may contribute to the improvement observed in the values of erythrocyte quality even though the extracellular environment remains unchanged in CKD [17]. With regard to this, we conducted preliminary investigation into whether epoetin beta (EPO) could also increase erythrocyte deformability. Using the same UNX-Thy1 experimental model, EPO (20 U/kg, 3 times/week) was administrated subcutaneously and erythrocyte deformability was assessed at 8 weeks after the start of the treatment. As observed with the C.E.R.A. treatment, EPO treatment also significantly improved erythrocyte deformability which was deteriorated in the UNX-Thy1 model rats (data not shown). Although further study is needed, we assume that the improvement in erythrocyte quality, including deformability, under the treatment of anemia may be observed as a class effect of ESAs.

The results leave room for several interpretations as to whether the alteration of erythrocyte quality is due to erythropoietin-dependent or -independent mechanisms. In regard to this, we assumed that the qualitative improvement in erythrocytes was mainly attributed to the hematopoietic effect, specifically the newly born erythrocytes. Interestingly, Vota et al. previously indicated that erythropoietin did not have a direct effect on the decrease of intracellular calcium content and phosphatidylserine externalization in erythrocytes [33]. Instead, they suggested that the beneficial effect of erythropoietin on erythrocytes may result from the inhibition of oxidative stress. Thus, not only the relative increase of newborn erythrocytes but also the suppression of oxidative stress by C.E.R.A. [14, 34] may contribute to improving erythrocyte deformability. Actually, in our experiment, the value of the d-ROMs oxidative stress marker at Week 18 was significantly increased in the plasma of the UNX-Thy1 + Vehicle group and tended to decrease in the plasma of the UNX-Thy1 + C.E.R.A. group (Fig. 2). Thus, both erythropoietin-dependent and -independent mechanisms might contribute to the improvement of erythrocyte quality.

In this study, there were some experimental limitations. For example, we only demonstrated the correlation between changes in erythrocyte deformability and intracellular calcium concentration. It seems informative to note that direct stimulation by calcium or calcium ionophores causes phosphatidylserine externalization in erythrocytes [33]. However, although ex vivo analysis of the effect of high calcium conditions or calcium antagonists on erythrocyte deformability would be valuable, the value of erythrocyte deformability quantified with our ektacytometer was dependent on the solvent (i.e., whole blood and medium for incubation of erythrocytes). Furthermore, an enormous quantity of erythrocytes is needed to adequately quantify erythrocyte deformability, and there is an experimental limitation on the amount of erythrocytes that can be gathered after ex vivo incubation with calcium. Therefore, to investigate the changes in erythrocyte deformability under several conditions of calcium content, analytical methods other than those using an ektacytometer may be required. For further elucidation of the causal dependence between erythrocyte deformability and calcium signaling, for example, other experimental models that can measure the high calcium levels in the circulation may be suitable. In addition, it will be informative to elucidate what kinds of erythrocytes contribute most to improving the values of erythrocyte quality. The detection and separation of erythrocytes based on their detailed cellular profiles, such as young and aged erythrocytes, will provide us further valuable information. Finally, the results in this study cannot be mechanically extrapolated into the clinical setting. Not only is it important to investigate whether the results observed in animal models will be also seen in humans but it is also important to explore what kinds of clinical benefits besides anemia treatment will be observed in ESA-treated patients. Although there are few systematic clinical studies, several previous studies have indicated that erythrocyte deformability might affect the prognosis and severity of some diseases associated with anemia [9, 35]. Although the underlying mechanisms have not yet been elucidated, deterioration of erythrocyte deformability may adversely affect blood rheology [10, 35]. Therefore, improvement of erythrocyte quality may be expected to offer additional advantageous effects to patients undergoing treatment for anemia. It is possible that clinical studies from the viewpoint of erythrocyte quality may shed light on additional benefits for patients on ESA therapy.

## Conclusion

In conclusion, we demonstrated that adequate C.E.R.A. treatment exerted a favorable effect not only on the increase in erythrocyte number (hematopoiesis) but also on the improvement of erythrocyte quality (deformability and life-span). Therefore, C.E.R.A. administered for the treatment of CKD-associated anemia may confer these therapeutic benefits on erythrocytes. Improvement of erythrocyte quality is expected to have an advantageous therapeutic effect in renal anemia. Further study will contribute to a better understanding of the therapeutic benefits of C.E.R.A. treatment in CKD-associated anemia.

## Abbreviations

ANOVA: Analysis of variance
C.E.R.A: Continuous erythropoietin receptor activator (epoetin beta pegol)
CKD: Chronic kidney disease
EI: Elongation index
EPO: Erythropoietin
ESA: Erythropoiesis-stimulating agent
FBS: Fetal bovine serum
Hb: Haemoglobin
MCV: Mean corpuscular volume
PBS: Phosphate buffered saline
RBC: Red blood cell
UNX-Thy1: Uninephrectomized anti-Thy1.1 antibody-induced progressive glomerulosclerosis
